# Drug resistant tuberculosis in Africa: Current status, gaps and opportunities

**DOI:** 10.4102/ajlm.v7i2.781

**Published:** 2018-12-06

**Authors:** Nazir Ismail, Farzana Ismail, Shaheed V. Omar, Linsay Blows, Yasmin Gardee, Hendrik Koornhof, Philip C. Onyebujoh

**Affiliations:** 1 Center for Tuberculosis, National Institute for Communicable Diseases, National Health Laboratory Services, Johannesburg, South Africa; 2 Department of Medical Microbiology, University of Pretoria, Pretoria, South Africa; 3 Department of Internal Medicine, University of Witwatersrand, Johannesburg, South Africa; 4 Africa Centres for Disease Control and Prevention, Addis Ababa, Ethiopia

## Abstract

**Background:**

The World Health Organization End TB Strategy targets for 2035 are ambitious and drug resistant tuberculosis is an important barrier, particularly in Africa, home to over a billion people.

**Objective:**

We sought to review the current status of drug resistant tuberculosis in Africa and highlight key areas requiring improvement.

**Methods:**

Available data from 2016 World Health Organization global tuberculosis database were extracted and analysed using descriptive statistics.

**Results:**

The true burden of drug resistant tuberculosis on the continent is poorly described with only 51% of countries having a formal survey completed. In the absence of this data, modelled estimates were used and reported 92 629 drug resistant tuberculosis cases with 42% of these occurring in just two countries: Nigeria and South Africa. Of the cases estimated, the majority of patients (70%) were not notified, representing ‘missed cases’. Mortality among patients with multi-drug resistant tuberculosis was 21%, and was 43% among those with extensively drug resistant tuberculosis. Policies on the adoption of new diagnostic tools was poor and implementation was lacking. A rifampicin result was available for less than 10% of tuberculosis cases in 23 of 47 countries. Second-line drug resistance testing was available in only 60% of countries. The introduction of the short multi-drug resistant tuberculosis regimen was a welcome development, with 40% of countries having implemented it in 2016. Bedaquiline has also been introduced in several countries.

**Conclusion:**

Drug resistant tuberculosis is largely missed in Africa and this threatens prospects to achieve the 2035 targets. Urgent efforts are required to confirm the true burden of drug resistant tuberculosis in Africa. Adoption of new tools and drugs is essential if the 2035 targets are to be met.

## Introduction

Global declines in tuberculosis incidence^1^ provide evidence that political commitment together with aggressive plans to curb the disease can make a difference. These efforts not only stopped the upward spiral of tuberculosis incidence but reversed the trend. The World Health Organization (WHO) has now set targets to end tuberculosis by 2035 and is directing efforts at accelerating the rate of decline, with the expectation of reducing the incidence rate by 90% and mortality by 95% compared to levels in 2015.^2^

Africa is home to over 1 billion people and is disproportionately affected by tuberculosis with 2.6 million of the 10.4 million global tuberculosis cases,^3^ making the continent a key geographical area for health interventions. Sub-Saharan Africa, in particular, saw rates rapidly escalate in the early 1990s due to a delayed response to the emergent HIV epidemic at the time.^4,5^ These failures resulted in incidence rates that are the highest in the world and have made the task to end tuberculosis even more challenging. Nonetheless, the tide has changed with the rapid expansion of anti-retroviral therapy resulting in sharp declines in HIV-associated tuberculosis incidence in countries in sub-Saharan Africa, thus offering a window of hope.^6,7,8^

Several factors threaten the potential to realise these targets, and key among these is drug resistant tuberculosis.^9^ Unfortunately, much like the global situation, drug resistant tuberculosis in Africa is largely missed with 93 000 cases estimated in 2016, while only 27 828 (30%) were diagnosed.^3^ Even when the diagnosis is made, only 59% achieve a successful treatment outcome.

We present a review of the current status of drug resistant tuberculosis in Africa using data from 2016 WHO global tuberculosis database,^10^ focusing on epidemiology, diagnostic tools and therapeutic options. We also highlight and discuss potential priority areas that require strengthening leading up to attaining the 2035 targets.

## Situation analysis of drug resistant tuberculosis in Africa

The best estimate of the burden of drug resistant tuberculosis in Africa requires population-based survey findings, as testing for all tuberculosis cases for rifampicin drug resistance is not done routinely in most countries. Only three countries – Mauritius, South Africa and Swaziland – had over 80% of cases tested for rifampicin (Table 1). Drug resistance surveys are thus the main source of data and are applied based on robust methodologies.^11^ Such surveys have however been completed for only 24 of 47 (51%) countries (Table 2). When restricted to the last five years, the figure is even lower at 23% (11/47). For 21 countries, no data are available, making planning a response much more challenging and potentially less effective. No country in Africa had a repeat survey in the five-year window recommended, and assessing trends was thus largely not possible based on survey data alone.

**TABLE 1:** Rifampicin resistance detection in Africa from policy to application.

Country	Population	Policy WRD as initial test	No. of Xpert MTB/RIF sites	Xpert MTB/RIF population (per million)	New incident tuberculosis cases	Rifampicin DST	Proportion of tuberculosis cases with rifampicin DST (%)
Mauritius	1 262 132	–	–	0.0	121	114	94
South Africa	56 015 473	Yes	207	3.7	237 045	215 696	91
Swaziland	1 343 098	Yes	28	20.8	3567	2915	82
Senegal	15 411 614	No	12	0.8	12 878	8934	69
Rwanda	11 917 508	Yes	47	3.9	5792	3849	66
Gambia	2 038 501	Yes	1	0.5	2498	1644	66
Seychelles	94 228	–	–	0.0	12	6	50
Mozambique	28 829 476	Yes	63	2.2	71 842	35 880	50
Ethiopia	102 403 196	No	138	1.3	125 836	56 509	45
Nigeria	185 989 640	Yes	318	1.7	97 279	39 819	41
Ghana	28 206 728	No	105	3.7	14 167	5359	38
Uganda	41 487 965	Yes	111	2.7	43 413	12 065	28
Kenya	48 461 567	Yes	130	2.7	76 335	20 884	27
Liberia	4 613 823	Yes	9	2.0	7105	1876	26
Sao Tome and Principe	199 910	Yes	1	5.0	188	40	21
Cabo Verde	23 439 189	No	2	0.1	247	50	20
Zimbabwe	16 150 362	Yes	116	7.2	27 353	5282	19
Guinea	12 395 924	No	10	0.8	12 639	2421	19
Malawi	18 091 575	No	42	2.3	15 516	2897	19
United Republic of Tanzania	55 572 201	Yes	68	1.2	64 609	9949	15
Mali	17 994 837	Yes	8	0.4	6776	1036	15
Gabon	1 979 786	No	2	1.0	5567	639	11
Cote d’Ivoire	539 560	No	13	24.1	21 357	2358	11
Democratic Republic of the Congo	78 736 153	No	60	0.8	130 596	13 273	10
Togo	7 606 374	No	1	0.1	2755	236	9
Burundi	10 524 117	Yes	6	0.6	7567	615	8
Equatorial Guinea	1 221 490	No	2	1.6	1428	105	7
Benin	10 872 298	No	6	0.6	3891	280	7
Burkina Faso	18 646 433	No	15	0.8	5677	376	7
Eritrea	4 954 645	No	15	3.0	2215	144	7
Cameroon	4 594 621	No	14	3.0	25 975	1541	6
South Sudan	12 230 730	No	2	0.2	10 770	536	5
Madagascar	24 894 551	No	5	0.2	29 001	1428	5
Namibia	2 479 713	Yes	25	10.1	8857	387	4
Congo	23 695 919	No	1	0.0	10 424	336	3
Chad	795 601	No	3	3.8	10 777	339	3
Niger	20 672 987	No	4	0.2	9921	287	3
Botswana	2 250 260	Yes	34	15.1	4803	104	2
Central African Republic	14 452 543	Yes	1	0.1	9968	206	2
Zambia	16 591 390	Yes	69	4.2	38 326	526	1
Angola	28 813 463	Yes	15	0.5	59 513	452	1
Comoros	5 125 821	–	–	0.0	163	1	1
Sierra Leone	7 396 190	No	6	0.8	14 114	60	0
Algeria	40 606 052	Yes	1	0.0	22 801	89	0
Mauritania	4 301 018	No	0	0.0	2359	8	0
Lesotho	2 203 821	Yes	22	10.0	7291	0	0
Guinea-Bissau	1 815 698	No	2	1.1	2226	0	0
Africa	1 019 920 181	21	1740	1.7	1 273 560	451 551	35

*Source*: WHO Global TB database.^10^

DST, drug susceptibility testing; WRD, WHO-endorsed rapid diagnostic.

**TABLE 2:** Comparison of rifampicin-resistant and multi-drug-resistant tuberculosis burden estimated with notified, ordered from highest to lowest estimated burden: 2016.

Country	RR/MDR estimated	RR/MDR estimated (low)	RR/MDR estimated (high)	RR/MDR notified	Missing RR/MDR (%)	Missing RR/MDR (low) (%)	Missing RR/MDR (high) (%)	Most recent DRS
Nigeria	20 000	12 000	29 000	1686	92	86	94	2010
South Africa	19 000	12 000	25 000	19 073	0	–59	24	2014
Democratic Republic of the Congo	7600	3900	11 000	709	91	82	94	2017
Mozambique	7600	4500	11 000	911	88	80	92	2007
Ethiopia	5800	3100	8500	700	88	77	92	2005
Angola	4300	1400	7300	167	96	88	98	–
Kenya	3000	1600	4400	326	89	80	93	2014
United Republic of Tanzania	2600	630	4600	196	92	69	96	2007
Cote d’Ivoire	2100	1100	3100	440	79	60	86	2017
Zambia	2100	1400	2900	180	91	87	94	2008
Uganda	1900	980	2900	489	74	50	83	2011
Zimbabwe	1900	1300	2600	572	70	56	78	2016
Cameroon	1600	1000	2200	176	89	82	92	–
Lesotho	1100	710	1400	–	100	100	100	2014
Namibia	960	740	1200	360	63	51	70	2015
Ghana	840	270	1400	107	87	60	92	2017
Chad	760	160	1400	45	94	72	97	–
Guinea	730	140	1300	178	76	–27	86	–
Sierra Leone	720	120	1300	13	98	89	99	–
Niger	660	140	1200	49	93	65	96	–
South Sudan	660	430	900	13	98	97	99	–
Swaziland	660	340	980	181	73	47	82	2009
Congo	640	390	900	29	95	93	97	–
Algeria	460	140	780	31	93	78	96	2002
Madagascar	440	81	790	40	91	51	95	2007
Senegal	440	280	610	64	85	77	90	2014
Liberia	430	46	820	92	79	–100	89	–
Burundi	420	270	580	80	81	70	86	–
Botswana	410	290	540	104	75	64	81	2008
Gabon	400	290	500	30	93	90	94	–
Malawi	400	130	670	66	84	49	90	2011
Mali	380	100	660	386	–2	–286	42	–
Burkina Faso	300	180	410	58	81	68	86	2017
Guinea-Bissau	200	17	390	37	82	–118	91	–
Central African Republic	180	0	410	57	68	–	86	2009
Eritrea	140	28	260	24	83	14	91	–
Mauritania	140	23	260	8	94	65	97	–
Rwanda	140	89	190	81	42	9	57	2015
Equatorial Guinea	110	55	170	32	71	42	81	–
Gambia	110	18	200	2	98	89	99	–
Togo	110	23	200	11	90	52	95	–
Benin	97	2	190	18	81	–800	91	2010
Comoros	45	9	81	1	98	89	99	–
Cabo Verde	29	9	48	0	100	100	100	–
Sao Tome and Principe	10	5	15	3	70	40	80	–
Mauritius	8	0	17	3	63	–	82	–
Seychelles	0	0	0	0	–	–	–	–
Africa	92 629	50 435	135 271	27 828	70	45	79	–

*Source*: WHO Global TB database.^10^

DRS, drug resistance survey; MDR, multi-drug resistant; RR, rifampicin resistant.

In light of the above limitations, WHO applies modelling using available data sources, including surveys.^12^ This serves as the best estimate of the burden of drug resistant tuberculosis. As modelling is based on selected assumptions, its use is not meant to replace the ideal of robust routine data. The estimated drug resistant tuberculosis burden is shown in Table 2, and countries are ranked from highest to lowest. Nigeria (20 000) together with South Africa (19 000) account for 42% (39 000/92 629) of the total estimated burden, and thus their achievements or failures regarding drug resistant tuberculosis control will have a great impact on the overall picture for Africa. When the next three highest burden countries – Democratic Republic of Congo (7600), Mozambique (7600) and Ethiopia (5800) – are added, the cumulative figure accounts for 65% (60 000/92 629) of the estimated burden of cases. This highlights the obvious heterogeneity of disease burden among African countries and the need for a targeted response rather than a generalised one.

An interesting modelling study was undertaken by Musa et al.^13^ using available data sources, including surveys and published studies in sub-Saharan Africa, to estimate the trends and regional prevalence of drug resistant tuberculosis. The region with the highest prevalence was in the south of Africa with 3.1% (2.1% – 4.2%) of tuberculosis cases being drug resistant. This was followed by central (2.1%; 1.1% – 3.0%), western (1.9%; 1.2% – 2.6%) and eastern (1.7%; 1.1% – 2.2%) regions of sub-Saharan Africa. These prevalence estimates correlate well with recent surveys in the region.

Another modelling study by Sharma et al.,^14^ which included four geographically diverse countries, predicted that there will be an increase in multi-drug resistant (MDR) tuberculosis across all four countries analysed and a decline in the relative contribution of acquired drug resistance. The MDR tuberculosis prevalence for South Africa come 2040 is predicted to be 5.7% (95% prediction interval 3.0–7.6) compared with 12.4% (9.4–16.2) for India, 8.9% (4.5–11.7) for the Philippines and 32.5% (27.0–35.8) for Russia. Although the estimated prevalence is higher in other parts of the world, this needs to be seen in the context of the high incidence of tuberculosis in sub-Saharan Africa, thus, single digit changes in prevalence constitute substantial changes in absolute numbers. Furthermore, the prediction of a global increase has implications for Africa, and the authors indicated that existing activities through the Green Light Committee will not be sufficient to change this trend.

The most recent survey in South Africa (2012–2014), compared to the previous one conducted just over 10 years earlier, showed an almost doubling of the rate in rifampicin resistance (1.8% to 3.4%)^15^ Also a similar increasing rate of resistance was observed in Botswana from 2.0% to 3.6% between 2002 and 2007–2008.^16^ The increase observed was primarily in new cases for both countries and driven by rifampicin mono-resistant tuberculosis, particularly in the South African survey. The increase in rifampicin resistant (RR) tuberculosis among new cases highlights the role of primary drug resistant tuberculosis transmission, which is likely to occur due to missed diagnostic opportunities when patients are not tested and treated for drug-resistant tuberculosis, or never reach health services. This has been the major obstacle in the era preceding the introduction of molecular diagnostics (Xpert^®^ MTB/RIF [Cepheid, Sunnyvale, California, United States] and line probe assays), which still continues to persist. Of importance as well is the higher rate of isoniazid mono-resistance (6.1%) compared to any rifampicin resistance (4.6%) in the 2012–2014 South Africa survey, with the former also associated with poorer treatment outcomes.

Comparing the estimated burden of drug resistant tuberculosis to cases notified, there is an alarming notification gap, as only 27 828 of the 92 629 estimated cases were notified. This suggests that 70% of cases in Africa are being missed. In addition, the proportion of missed RR and MDR tuberculosis cases are estimated to be above 50% for 43 of the 47 African countries (Table 1). It should be noted that the confidence intervals are relatively wide, yet using the lower estimate the gap is 45%. The extremely drug resistant tuberculosis estimates are not available for comparison; however, a total of 1092 extremely drug resistant tuberculosis cases were notified, of which South Africa reported 89% (967) of all extremely drug resistant tuberculosis cases on the continent.^17^ This disproportionality is most likely due to lack of capacity to diagnose extremely drug resistant tuberculosis in Africa. Only 53% of RR and MDR tuberculosis cases in Africa had second-line drug susceptibility testing performed, and 47% (22/47) of member states do not have a laboratory that can perform second-line resistance testing (Table 3).

**TABLE 3:** Second-line resistance detection in Africa from policy to application.

Country	Policy on universal DST	No. of LPAsl sites	No. of DST-SLT sites	Confirmed RR/MDR tuberculosis	No. of RR/MDR-tuberculosis with SLT	Proportion of RR/MDR- tuberculosis with SLT (%)	NRL ISO15189 accredited
Benin	No	1	1	18	18	100	Yes
Cameroon	No	1	1	176	176	100	Yes
Gabon	No	1	1	30	30	100	Yes
Gambia	No	0	0	2	2	100	Yes
Swaziland	No	0	1	181	181	100	Yes
Equatorial Guinea	Yes	–	–	32	32	100	–
Madagascar	Yes	0	1	40	40	100	No
Mauritius	Yes	–	–	3	3	100	–
Sierra Leone	Yes	0	0	13	13	100	No
Mozambique	Yes	0	1	911	868	95	Yes
Niger	Yes	1	1	49	34	69	No
Cote d’Ivoire	No	0	0	440	300	68	No
Burundi	No	0	1	80	52	65	Yes
Kenya	Yes	1	5	326	204	63	Yes
South Africa	Yes	7	7	19 073	11 903	62	Yes
Zimbabwe	No	2	1	572	301	53	Yes
United Republic of Tanzania	No	–	1	196	97	49	Yes
Rwanda	No	1	1	81	35	43	No
Algeria	–	1	1	31	13	42	Yes
Senegal	Yes	1	1	64	22	34	No
Democratic Republic of the Congo	No	1	2	709	223	31	No
Uganda	Yes	0	4	489	101	21	Yes
Namibia	No	1	1	360	54	15	Yes
Guinea	Yes	1	1	178	26	15	No
Ethiopia	Yes	1	1	700	28	4	Yes
Mali	No	0	2	386	6	2	Yes
Chad	No	–	–	45	0	0	–
Congo	No	1	1	29	–	0	Yes
Eritrea	No	0	0	24	0	0	Yes
Ghana	No	0	0	107	0	0	Yes
Guinea-Bissau	No	0	0	37	–	0	No
Malawi	No	–	–	66	0	0	Yes
Sao Tome and Principe	No	0	0	3	0	0	No
South Sudan	No	0	0	13	–	0	No
Angola	Yes	0	3	167	0	0	No
Botswana	Yes	1	1	104	0	0	Yes
Burkina Faso	Yes	1	0	58	0	0	No
Central African Republic	Yes	0	0	57	0	0	No
Nigeria	Yes	2	2	1686	–	0	Yes
Zambia	Yes	1	3	180	0	0	Yes
Comoros	–	0	0	1	0	0	No
Liberia	–	0	0	92	0	0	Yes
Mauritania	–	0	0	8	0	0	No
Togo	–	0	0	11	0	0	No
Cabo Verde	No	2	2	0	0	–	Yes
Seychelles	No	–	–	0	0	–	–
Lesotho	Yes	0	0	–	–	–	Yes
Africa	19	29	48	27 828	14 762	53	26

*Source*: WHO Global TB database.^10^

DST, drug susceptibility testing; NRL, National Reference Laboratory ; SLT, second-line testing.

**FIGURE 1:**
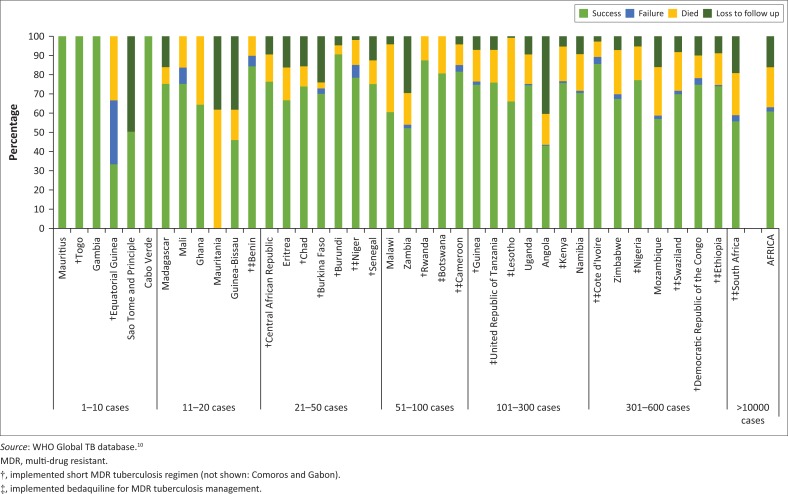
Multi-drug-resistant tuberculosis outcomes by country grouped by size of cohort: 2014. Countries with no data or no reported cases are not shown.

Mortality reduction is another important target of WHO’s 2035 End TB Strategy. Missed cases contribute to mortality directly when cases are not detected through current programmes, or indirectly due to late presentation or testing for resistance being unavailable. The overall MDR tuberculosis mortality for the 2014 Africa cohort (*N* = 16 231) was 20%, while treatment success was only 58% (Figure 1). The situation was far worse for the extremely drug resistant tuberculosis cohort in 2014 (*N* = 623) with mortality at 42% and treatment success at only 27%. Outcomes for the 2014 cohort of MDR tuberculosis by country are shown in Figure 1.

Reviewing the diagnostic landscape for drug resistant tuberculosis in Africa, encouraging signs are noted with 45% (21/47) of countries having progressive policies, which include the use of the Xpert MTB/RIF assay as the initial WHO-endorsed rapid diagnostic test (Table 1). Comparing policy to practice, clear gaps emerge. Across the continent only 35% of newly diagnosed tuberculosis cases had a rifampicin drug susceptibility test performed (Table 1), implying that primary drug resistant tuberculosis cases are largely being missed. Rifampicin drug susceptibility testing was available for less than 10% of new cases in 23 countries. In contrast, Mauritius (94%), South Africa (91%), Swaziland (82%), Senegal (69%) and Rwanda (66%) were ranked as the top five countries with high coverage for rifampicin drug susceptibility testing among notified cases. On the continent, the Xpert MTB/RIF is available in 43/47 (91%) countries, comprising 1740 testing sites (Table 1). Adjusted for population size, there is large variability ranging from less than 1 to 24 sites per million people. Albeit a crude measurement, it does highlight important gaps in coverage for many countries. However, coverage of drug susceptibility testing does not directly imply utilisation, which is likely to be even poorer.

Policies on universal drug susceptibility testing are also lacking with 19 (40%) of the 47 countries having a policy in place (Table 3). The GenoType MTBDR*sl* (Hain Life Sciences, Nehren, Germany) for second-line drug susceptibility testing is available in 20 of 47 (43%) countries at 29 sites (Table 3). This proportion is unsurprisingly low as the GenoType MTBDR*sl* has only been recently endorsed to address gaps in second-line drug susceptibility testing. Overall, 28 (60%) countries have second-line drug susceptibility testing available, using either line probe assay or phenotypic drug susceptibility testing (Table 3). Availability of second-line drug susceptibility testing has, however, not translated into practice with only 43% (12/28) of these countries testing more than half their notified rifampicin-resistant tuberculosis cases. However, these percentages are not truly reflective, as they are based on the number of detected rather than estimated cases.

Another important issue with respect to laboratories is quality assurance. Among all African countries, 26 (55%) national reference laboratories (NRLs) reported having an ISO 15189 accreditation status. Although this does seem encouraging, this reporting may not be a true reflection of the situation, as the reference laboratories that are formally recognised by SLMTA^18^ as being accredited based on external evaluation are the NRLs of Ethiopia, Mozambique, South Africa and Uganda. ISO 15189 accreditation should be a basic requirement for this level of laboratory. In the absence of accreditation, participation in external quality assurance programmes provides a basic measure of quality evaluation and competence while countries progress towards accreditation and has been introduced for high burden countries through the WHO.

Apart from diagnostics, treatment is an essential component of tuberculosis control as well and affects a country’s ability to reach the WHO End TB Strategy targets. Drug resistant tuberculosis treatment is often prolonged and uses less effective regimens with more adverse events compared to first-line treatment. The number of cases reported on treatment per country varies (Figure 1), with all but one treating less than 600 cases in a year (South Africa: > 10 000). The treatment success overall in Africa was 59%, 20% died and 16% were lost to follow-up (Appendix 1). The introduction of the short-course regimen is a major improvement and, in 2016, 36% (17/47) of countries used this patient friendly regimen (Figure 1). Countries leading the implementation of this regimen were Democratic Republic of Congo (555), Cote d’Ivoire (349) and Cameroon (135) with 31%, 68% and 100% of RR and MDR tuberculosis patients in these countries, respectively also having second-line drug susceptibility testing performed. Thus, adoption of the short-course regimen is not strictly linked to second-line drug susceptibility testing; however, in light of the recent rapid WHO guidance, the need for early identification of fluoroquinolone resistance is important. The top three countries with at least 50 cases or more and having the highest treatment success were countries implementing the short-course regimen: Burundi (90%), Rwanda (88%) and Cote d’Ivoire (85%). Bedaquiline is a new and welcome addition to drug resistant tuberculosis management and is reported to be highly effective.^19,20^ It is currently being used in 11 African countries as well as in South Africa where its use is on a large scale (Figure 1). This new agent also offers a potential for scale-up in addressing poor treatment outcomes for drug resistant tuberculosis.

## Towards ending drug resistant tuberculosis in Africa

Drug resistant tuberculosis poses a major hurdle to achieving the WHO End TB Strategy targets. Acting early and decisively will be a determining factor in either future success or failure. Encouragingly, the past 5 years have seen new diagnostic technologies and treatment options become available, as well as strong global political commitment to end tuberculosis. Despite the obstacles threatening the realisation of the WHO End TB Strategy targets in Africa, there are equally effective tools available for achieving success.

Primary among the urgent needs is a clear understanding of the burden of tuberculosis and drug resistant tuberculosis, which will greatly impact planning and efficient resource allocation, a key issue in resource-constrained environments. Progress has been made with just over half of the countries ever having completed a survey. However, there are still large gaps in the data available and these need to be urgently addressed. Furthermore, in order to assess progress and respond appropriately, trend data is essential. The use of routine data reported to the WHO has added benefits, but concerns about accuracy and completeness result in this information being treated with circumspection. Modelled estimates using this routine data would also be impacted as a consequence.

An alternative approach to address gaps is the use of sentinel surveillance for tracking annual trends in strategically selected sites. This is a potential hybrid solution, which is being considered by the HIV programme.^21^ Such a system has been used in South Africa for tuberculosis, and unpublished data do support the value of such an approach. This approach can be achieved with far fewer resources and has the potential to strengthen existing routine systems and serve as a starting point for replication of developed sites in other areas. Ensuring routine standardised algorithms detecting rifampicin and second-line resistance is the ideal and should be facilitated in the era of the Xpert MTB/RIF and GenoType MTBDR*sl*. A few countries have shown high uptake of rapid diagnostic tools for early rifampicin and second-line resistance detection. These efforts should be standardised to ensure data comparisons between countries and regions of Africa.

Another key concern is missing cases, which are estimated to be equal to or greater than the notified burden. Although the numbers are staggering, modifying current diagnostic algorithms to adopt new technologies could dramatically reduce this gap in a short period of time and can be seen in some countries already. The utilisation of the Xpert MTB/RIF has been lacklustre on the continent, with many countries limiting its use to selected cases and thus diminishing the impact of this critical molecular diagnostic platform. This not only reduces the detection of tuberculosis by approximately 10% – 20%,^22^ but also means that by design, rifampicin resistance is missed almost completely in the vast majority of tuberculosis cases. It should be noted that although the prevalence of drug resistant tuberculosis is higher in previously treated cases compared to new cases, the absolute number of drug resistant tuberculosis patients among previously treated cases is far lower than the numbers in new tuberculosis cases. For the 2015 cohort, 1 200 078 new and relapsed drug resistant tuberculosis cases were notified, of which only 38 059 were previously treated cases.^3^ The net effect is a similar burden in absolute numbers of drug resistant tuberculosis between new and previously treated cases. This is because previously treated cases, despite having a much higher prevalence of drug resistant tuberculosis cases, contribute only to a small part of the total TB burden.

Aggressive scale-up of the use of rapid molecular diagnostic tools (e.g. Xpert) as the primary test will be key to finding missing drug resistant tuberculosis cases and is all the more urgent as primary transmission is a major contributor to the propagation of the disease.^15,22^ Missing resistance may result in poorer outcomes, and further compromises the health and well-being of the population at large. In addition, stigma and lack of access to health services are known obstacles to care and likely contribute to missing cases. Some of these cases will most likely end up at a health facility and the unavailability of quality diagnostic technologies would lead to a catastrophic treatment failure. Several countries other than South Africa have taken the approach of using the Xpert MTB/RIF as the primary test for the detection of tuberculosis and drug resistant tuberculosis, and thus models for implementation are available. The South African approach uses a central model with important emphasis on logistics, which has worked well. In regions where transport infrastructure is limited, the use of the GeneXpert Omni (Cepheid, Sunnyvale, California, United States) could be an effective solution. The greater focus now is on multiplex diagnostic platforms that will also facilitate integration at different levels of the health system, thereby providing improved diagnostic yield. Irrespective of the approach, careful planning and budgeting will be needed in order to achieve the necessary returns.

A second issue relates to second-line testing. While this is being achieved in several African countries with good overall coverage among notified RR tuberculosis cases (Table 3), it is often a challenge in most settings across Africa with 43% (20/47) of member states having no coverage. As the overall reported burden of drug resistant tuberculosis is not particularly high in many countries, the need and operational feasibility to set up such systems may be better served through regional collaboration. There are three WHO-certified supranational reference laboratories in Africa^24^ and several NRLs that have adequate capacity to provide such services. While this would lead to increase in volumes of second-line testing, it will also ensure operational efficiency and allow skills to be developed and sustained. Linked to such services is also the need for supporting surveys on the continent.

The three supranational reference laboratories are limited by capacity to provide for the large needs of the continent. Expanding the number of supranational reference laboratories is required and NRLs with potential should be supported to achieve this status. The number of NRLs complying with ISO 15189 is still unacceptably low, considering that NRLs are expected to be the standard against which sub-national laboratories are compared. The introduction of the Strengthening Laboratory Management Toward Accreditation programme has seen 54 laboratories achieve accreditation by the end of 2017 over a 5-year period;^25^ however, only two were national tuberculosis reference laboratories. Prioritisation of NRLs to achieve accreditation should be a short-term goal using Strengthening Laboratory Management Toward Accreditation. The emergence of the new Africa Centres for Disease Control and Prevention portends hope through the introduction and implementation of the regional integrated laboratory and surveillance network hosted through the five Regional Collaborating Centres in the five geographical sub-regions of the African Union. It is anticipated that these centres, and the associated network, can be used to improve and strengthen diagnostic capabilities within the Africa region.

The continuum of care requires not only good diagnostics but also effective early treatment. Successful outcomes among MDR tuberculosis patients are exceptionally low with death and loss to follow-up being common endpoints. These are complex issues underpinned by late presentation, poor access to services and overburdened health services. Treatment options are another important determinant of patient outcomes. Therapy is often prolonged; however, a short-course regimen has shown promise in reducing the loss of patients to follow-up. The highest success rates observed in Africa were among those applying this approach and, importantly, a large evidence base for this policy came from Africa and confirms its value.^25^ The introduction of the short regimen requires greater impetus to ensure it is accessible and widely used, as currently only 36% of countries have introduced it.

More positive news out of Africa is the use of bedaquiline, with significant reductions observed in mortality for both MDR and XDR tuberculosis cases using this agent.^26^ This too is a timely improvement in addressing urgent issues in the management of drug resistant tuberculosis. South Africa has scaled up its use, but it has only recently been introduced in 11 countries. Time lost due to slow scale-up will result in preventable deaths and further transmission. Additionally, skills in dealing with complex drug interactions and adverse events are available, particularly in South Africa, and South-South collaboration can facilitate the safe and effective introduction of the new agents. The availability of a second agent – delamanid – is another promising advancement and opens the way for potentially effective combination therapy to be standardised for XDR tuberculosis treatment. This would simplify management of these complex cases, especially for areas where skills may be lacking. The Global Drug Facility is an important mechanism for access to new drugs for the treatment of drug resistant tuberculosis in Africa and can be utilised to improve the management of drug resistant tuberculosis.

As highlighted, South-South collaborations are important, and findings reported by Cain et al.^27^ demonstrate the impact of migration and management of drug resistant tuberculosis. These authors showed that a large and increasing case load of MDR tuberculosis patients from specific areas of Somalia crossed borders to Kenya, seeking care due to lack of services. Migration is an important contributor to missing cases and may lead to poor outcomes. Similar migratory behaviour has been documented in other regions on the continent related to employment seeking in the mining sector.^28^ Dealing with migration and the continuum of care requires common standards of care to be available, good communication and referral mechanisms, as well as a unique identifier to link patients across countries and regions. Although this issue has received some attention from the regions, the impetus has been slow and coordination weak. The launch of the Africa Centres for Disease Control as well as Prevention and its Regional Collaborating Centres and target national public health institutes does offer a potential coordination mechanism and should be prioritised. The Innovation in Laboratory Engineered Accelerated Diagnostics project is a new initiative and will use biometrics^29^ as the unique patient identifier across regions. If successful, it will provide a model to allow patients to be managed through care irrespective of where they are diagnosed.

Ending tuberculosis and specifically drug resistant tuberculosis cannot be fully realised without dealing with HIV infection and disease. HIV/AIDS is a major contributor to the burden of both tuberculosis and drug resistant tuberculosis, and current efforts at also ending HIV are encouraging. Achieving viral suppression at a population level is an important tuberculosis prevention strategy and will likely lead to continued declines in HIV-associated tuberculosis and drug resistant tuberculosis. This would, however, result in a relatively higher proportion of HIV-negative tuberculosis and drug resistant tuberculosis. This group is less likely to die and, consequently, can transmit for longer periods. Thus, missing such cases will have long-term consequences, and messages to ensure this group is also investigated will be increasingly important. Another issue that should be appreciated is that the burden of tuberculosis and drug resistant tuberculosis has for many years exceeded the existing medical care capacity, leading to failures in health delivery. In spite of this, the declines seen offer a window of hope where the developed capacity and burden may align once again. Thus, the need to maintain funding and capacity during this period is needed if we are to end tuberculosis by 2035.

### Limitations

It is important to contextualise the limitations of this analysis. The data used were taken from the WHO global tuberculosis database, which derived data from countries through unverified self-reporting. Countries are usually given time to verify their data before submission and, in addition, anomalies identified by WHO result in queries sent back to countries for checking before the data are accepted. Additionally, the estimates included are based on established mathematical models adopted for use by WHO and are inherently influenced by the assumptions applied to the model. The wide confidence intervals account for the uncertainty in deriving these estimates.

### Conclusion

Drug resistant tuberculosis is difficult to manage even in the best of environments and is likely to pose a major challenge for Africa as it works toward achieving the WHO End TB Strategy targets. Any delays in addressing drug resistant tuberculosis will mean lost ground, which will make the challenge even greater. Our ability to end tuberculosis and, specifically, drug resistant tuberculosis, by 2035 will require a major uphill effort, but it is achievable given the right strategic focus complemented by strong leadership and adequate resources. The adage ‘know your epidemic, and know your response’ serves as a guiding principle leading up to 2035 and we have provided detailed data clearly highlighting areas of success and failure. It is clear that the burden is highly heterogeneous, and focusing on key countries will be greatly rewarding, if available resources are used wisely. Gaps in data are also large and certainty needs to be achieved on the true burden of tuberculosis and drug resistant tuberculosis throughout the continent. Experience gained in addressing the deficiencies identified here could influence prioritisation within the tuberculosis control programme in the future. The advent of new and improved diagnostics constitutes a major advancement, although adoption has not been aggressive enough in many parts of Africa, and this needs greater impetus. Despite shortcomings as a continent, African countries have played a leading role for both the evaluation of drug resistant tuberculosis diagnostic tools and treatment options, which include the short regimen and bedaquiline. These findings need to move to scale rapidly for us to accelerate progress in dealing with drug resistant tuberculosis and ultimately end the disease.

## Appendix 1

**TABLE 1-A1:** MDR-TB outcomes in Africa: 2014.

Number of cases	Country	RR/MDR-tuberculosis cohort 2014	Treatment success	Treatment failure	Died	Loss to follow-up	% Successful outcome	% Died	% Lost to follow-up
1–10 cases	Mauritius	1	1	0	0	0	100	0	0
	Togo†	1	1	0	0	0	100	0	0
	Gambia	2	2	0	0	0	100	0	0
	Equatorial Guinea†	3	1	1	1	0	33	33	0
	Sao Tome and Principe	4	2	0	0	2	50	0	50
	Cabo Verde	5	5	0	0	0	100	0	0
11–20 cases	Madagascar	12	9		1	2	75	8	17
	Mali	12	9	1	2	0	75	17	0
	Ghana	14	9	0	5	0	64	36	0
	Mauritania	14	0	0	8	5	0	57	36
	Guinea-Bissau	15	6	0	2	5	40	13	33
	Benin†‡	19	16	1	2	0	84	11	0
21–50 cases	Central African Republic†	21	16	0	3	2	76	14	10
	Eritrea	24	16	0	4	4	67	17	17
	Chad†	25	14	0	2	3	56	8	12
	Burkina Faso†	34	23	1	1	8	68	3	24
	Burundi†	41	37	0	2	2	90	5	5
	Niger†‡	46	36	3	6	1	78	13	2
	Senegal†	48	36	0	6	6	75	13	13
51–100 cases	Malawi	53	31	0	18	2	58	34	4
	Zambia	68	35	1	11	20	51	16	29
	Rwanda†	80	70		10	0	88	13	0
	Botswana‡	86	63	0	15	0	73	17	0
	Cameroon†‡	91	74	3	10	4	81	11	4
101–300 cases	Guinea†	123	92	2	20	9	75	16	7
	United Republic of Tanzania‡	143	108	0	25	10	76	17	7
	Lesotho‡	152	98	0	48	2	64	32	1
	Uganda	214	153	1	31	20	71	14	9
	Angola	249	107	2	39	101	43	16	41
	Kenya‡	251	180	1	43	13	72	17	5
	Namibia	266	186	4	49	26	70	18	10
301–600 cases	Cote d’Ivoire†‡	311	265	13	23	10	85	7	3
	Zimbabwe	381	193	6	66	21	51	17	6
	Nigeria‡	423	314	1	72	21	74	17	5
	Mozambique	439	218	8	95	63	50	22	14
	Swaziland†‡	444	310	8	88	38	70	20	9
	Democratic Republic of the Congo†	448	334	16	51	46	75	11	10
	Ethiopia†‡	557	388	3	85	49	70	15	9
>10000 cases	South Africa†‡	11 111	6042	336	2410	2105	54	22	19
	Africa	16 231	9500	412	3254	2600	59	20	16

*Source*: WHO Global TB database.^10^

Countries with no data or no reported cases are not shown.

† , implemented short MDR tuberculosis regimen (not shown: Comoros and Gabon).

‡ , implemented bedaquiline for MDR tuberculosis management.

MDR, multi-drug resistant; RR, rifampicin resistant.
